# Devastating Flood in Nepal Amid a Humanitarian Crisis: A Call for Action

**DOI:** 10.31729/jnma.8846

**Published:** 2024-12-31

**Authors:** Bibek Giri, Minal Jehan, Mubashir Mohiuddin, Ahmad Noor, Ahmed Sheraz

**Affiliations:** 1 Research Institute for Collaborative Development, Kathmandu, Nepal; 2 Karachi Medical and Dental College, Karachi City, Sindh, Pakistan; 3 Dow University of Health Sciences, Karachi City, Sindh, Pakistan; 4 Allama Iqbal Medical College, Lahore, Panjab, Pakistan

**Keywords:** *basic needs*, *disaster*, *diseases*, *flood*, *Nepal*

## Abstract

Floods have been a major problem in Nepal for a long time. Many lives have been lost due to the flood and people are still suffering from its effect. People are homeless and facing basic problems like food, and shelter. There are rising cases of waterborne diseases due to contaminated water. Providing healthcare facilities in this situation is additional challenge to the government. This has been a challenging issue for the Nepal government, and that need to be stop. Therefore, in this paper, we discussed the various methods, and strategies to tackle this problem in the future.

## INTRODUCTION

Floods are among the most destructive natural disasters, triggered by ocean tides, heavy rainfall, or sudden lake outbursts.^1^ They cause significant loss of life and property globally, with underdeveloped nations suffering the most.^2^ Nepal, the second highest country at risk of floods in South Asia^3^ and lies in 11^th^ position on disaster risk in the world^4^ faces persistent flooding, especially during the monsoon season from June to September. Floods have caused thousands of deaths, affected millions, and resulted in substantial economic losses.^4^ This paper examines the impact of floods on Nepal's infrastructure and population, highlighting the urgent need for effective disaster management strategies.

## EFFECT OF FLOODING AND CHALLENGES

The 2024 floods in Nepal, following the rainy season, have had a devastating impact on the country's infrastructure, environment, and economy. Intense rainfall and flooding triggered landslides that destroyed roads, bridges, and utilities, particularly in the central provinces of Gandaki and Bagmati.^5^ This disruption has severely affected travel, communication, and rescue operations.^6,7^ The floods have also caused significant damage to Nepal's natural environment, inundating forests and national parks, displacing wildlife, and destroying habitats. The resulting loss of vegetation has led to increased soil erosion and barren land. These issues are exacerbated by climate change, which is altering rainfall patterns across South Asia and increasing the frequency of extreme weather events.^6-8^

Nepal's agriculture sector, crucial to its economy, faces severe long-term consequences. Crops have been destroyed, arable land eroded, and irrigation systems damaged, threatening food security and farmers' livelihoods.^6^ The depletion of essential natural resources, such as soil and water, has lasting impacts on agricultural productivity and sustainability. Economically, the floods have caused millions of dollars in damage to infrastructure, homes, and businesses. The disruption of key economic activities, especially in agriculture^6^ and tourism, has further exacerbated financial challenges for individuals and the nation, leading to increased poverty and hardships.

Thousands of people have been displaced, seeking shelter in temporary camps.^6^ This displacement has significant socio-economic consequences, including loss of property, family separation, and interrupted education for children. Displaced populations are more vulnerable to exploitation and violence, and the lack of stable housing and employment worsens their situation.^7^ The floods have also led to an increase in waterborne diseases, such as cholera, typhoid, and dysentery, due to contaminated water sources. Stagnant water has become a breeding ground for mosquitoes, increasing the incidence of malaria and dengue fever. These health risks are compounded by the destruction of healthcare facilities and shortages of medical supplies, making it difficult to prevent and treat diseases effectively.^7^

Providing healthcare during this crisis has been challenging. Damaged infrastructure, obstructed access to medical facilities, and the displacement of healthcare workers have limited available medical care. Increased demand for health services, coupled with limited resources, has strained the healthcare system, making it difficult to address the urgent needs of the affected population.^7^ Relief and recovery operations face numerous obstacles, including logistical challenges, lack of coordination, and limited resources. Damaged infrastructure hampers the delivery of aid, and fragmented communication networks complicate coordination efforts. The absence of comprehensive data on disaster impacts impedes effective planning and response. Often, relief efforts are reactive rather than proactive, highlighting the need for better disaster preparedness and resilience-building strategies.^8,9^

## HUMANITARIAN CRISIS UNFOLDING IN NEPAL

Nepal has chosen to fight the destructive floods that occur during monsoon seasons, inflicting landslides, fatalities, and destruction in many of the nation's districts. The flood-prone regions of the Terai districts—Banke, Bardiya, and Kailali—along the flood plains of the Karnali, Babai, and West Rapti rivers had to be the most badly impacted.^5^ The Terai region's high reliance on agriculture and vulnerable terrain make flood threats more severe.^5^ Thousands of homes have been damaged or destroyed, thereby leaving families without shelter. Farmlands have been flooded, and food security or livelihoods are under threat.^5^ Many routes have been blocked by landslides that have happened in mountainous places, and parts of the country have been inundated. Heavy rain could cause more flooding in low-lying areas and urban regions with poor drainage. Areas near large reservoirs or rivers may experience flash flooding, and landslides are likely in rain-saturated hilly regions.

The children, elderly and pregnant women are more vulnerable to flood. Children would suffer from waterborne diseases and malnutrition; the elderly and pregnant women require special medical attention that may not be reachable during a disaster.^10^ The worst affected are the families living in informal settlement and fragile housing along the riverbanks, as they have no better way of resistance and recovery from the floods.^5^ Flood damage, evacuations, and employee absences can disrupt Local business in affected area due to affecting their ability to function and serve their customers. Flooding could increase disease outbreaks, as stagnant water can breed mosquitoes and bacteria, raising insect- and water-borne diseases. During floods, water from industrial sites, sewer systems, and septic tanks can mix with floodwaters, leading to Contamination. This contaminated water poses significant health risks because it can carry harmful chemicals, pathogens, and waste. Exposure to such water can result in serious illnesses, including gastrointestinal infections, skin diseases, and respiratory problems.^11^

The populations affected by this disaster identified shelter, food, clean water, and medical care as their most essential needs. There is an urgent need for emergency shelter and assistance materials because many of them lost their houses and belongings. Safe drinking water was not guaranteed during floods.^12^ With limited supplies of medications and equipment to heal wounds and prevent disease outbreaks, medical facilities were overburdened.^12^ In the days ahead, authorities may issue mandatory evacuation orders for communities at risk of flooding. Large-scale flooding or landslides might affect utility networks, causing disruptions to telecommunications and energy services.^11^ It has undoubtedly been difficult to provide aid to isolated and vulnerable groups. Due to landslides and damaged infrastructure, the majority of the areas impacted by the water are inaccessible.^5^ Travel may be impacted if bridges, railroads, and roads become impassable due to floodwaters and debris flows. Ponding on roadways can make driving on highways dangerous.^11^ Under such circumstances, leaving nobody behind is itself a collaborative task of the government, humanitarian response teams, and the local administration to reach out to such isolated communities.^12^

## GOVERNMENT RESPONSE AND COMMUNITY ENGAGEMENT

Although the Nepalese government's response to the devastating floods has been difficult, it has also laid out some positive actions. It was crucial to activate humanitarian clusters and conduct preliminary, quick assessments.^13^ However, the government's ability and resources are already overextended due to the disaster's scope, making it impossible to efficiently serve every impacted community. Damage to vital infrastructure, like as roads and bridges, has made it extremely difficult to provide help to isolated locations.^13^-^14^ The lack of cooperation among national, provincial, and municipal authorities has also contributed to delays and gaps in aid. In the reaction attempts, those marginalized groups—most notably the Dalits—may also be disregarded.^13,15^

The flood response in Nepal has been greatly aided by international organizations. UNICEF has assisted with early recovery efforts and provided emergency relief supplies. Six Resources and personnel have also been assigned by other UN agencies, non-governmental organizations, and foreign governments to address the predicament of impacted populations.^15,16^ Nepal's neighbors, especially India, have been incredibly supportive, offering cross-border relief efforts and emergency assistance. Search teams, relief supplies, and the rebuilding of vital infrastructure are all examples of India's excellent response.^15^ This regional cooperation was of much importance to address the humanitarian crisis.

Although it is still ongoing, collaboration between the government, foreign organizations, and local populations has been crucial. To enhance resource allocation and data collection, the government has created new structures. However, reaching the most disadvantaged communities continues to be a challenge in the actual world.^13,16^ Engaging local organizations and community leaders keep aiding in adjusting relief operations to meet the specific requirements of the impacted population. However, more can be done to engage the community and their active participation throughout the process.^13,14^

## CALL TO ACTION

Floods and landslides in the small Himalayan nation of Nepal have caused significant disruption, necessitating immediate and effective management strategies. Immediate steps should include implementing existing evacuation plans and designating safe zones. Emergency teams need to be deployed, ensuring a steady supply of food, water, and hygiene kits, ideally delivered by boats and helicopters for swift action. The government should ban passenger buses in areas with flood warnings, a measure that should remain in place until the rains subside. As the rains are expected to move from different regions to all over the nation, relocating residents from landslide-prone areas should be a top priority. This can be facilitated using satellite technology for accurate meteorological monitoring and SMS-based alerts are recommended over other communication methods due to their higher effectiveness. Similarly, flood forecasting and warning systems are universally recognized as effective methods for flood management. The Department of Hydrology and Meteorology (DHM) is actively developing flood anticipation and warning systems for Nepal's major rivers, aiming to reduce the impact of such disasters.^5^

Disaster management task forces play a vital role in future crises, focusing on protecting vulnerable populations such as the elderly, pregnant women, and children. Despite the political instability of the nation, where the coalition government is struggling to maintain order, a unified appeal for financial aid and technical expertise is essential to address this natural disaster. Partnerships between nonprofit organizations and private companies should be encouraged to provide essential resources like food and shelter. Long-term management strategies could involve imposing fines or banning construction in floodplain areas prone to heavy rainfall. Increasing greenery by planting more trees and creating parks can help absorb excess water from heavy rains. To combat ongoing soil erosion, reforestation programs should be promoted. Given the global water scarcity, implementing rainwater harvesting systems is crucial for water conservation.

## CONCLUSIONS

Considering that last monsoon season caused the displacement of around 6,000 households and Nepal's geographical location makes it vulnerable to global climate change, it's crucial to handle the current monsoon season with a multifaceted approach. Every year the flood has been serious issues in Nepal. For future purposes, the government should fund research projects to develop plans concerning Nepal's specific needs. The Natural Disaster and Management Authority (NDMA) should be kept in the loop for timely assistance. All drainage systems should be cleared, and several portable pumps should be deployed to be used when needed. To build resilience against devastating events like floods, there is no better strategy than unified international assistance. The Nepalese government's response to the floods has been marked by challenges, though some commendable steps have been taken with the help of aid from international organizations and neighboring countries. Enhanced coordination, data-driven decision making, and community involvement would be vital for reversing the prevailing state of humanitarian crises and building resilience in the long term.
